# Supplementary material for: Intrinsically disordered RNA-binding motifs cooperate to catalyze RNA folding and drive phase separation
Source: Nucleic Acids Res. 2024 Nov 18;52(22):14205–28. doi: 10.1093/nar/gkae1107 (PMC11662656; doi:10.1093/nar/gkae1107)
Supplement: gkae1107_Supplemental_Files [file gkae1107_supplemental_files.zip › Niedner_PUN_Supplementary_Information.pdf]

Supplementary Information – Niedner-Boblenz et al.

A Micro-array analysis of RNAs immuno-precipitated with TAP-Loc1p

Gene	Mean	Function
RDN58-1	3.803	5.8S rRNA
YLR161W	3.707	uncharacterized ORF in proximity to rDNA
RDN5-1	3.367	5S rRNA
YLR159W	3.180	uncharacterized ORF in proximity to rDNA
YKL084W	2.080	HOT13; mitochondrial intermembrane space protein
YHL041W	2.057	dubious ORF unlikely to encode a functional protein
YLR119W	2.007	SRN1; component of ESCRT-I; perhaps involved in RNA export
YCR018C	1.977	SRD1; involved in processing of pre-rRNA to mature rRNA
YEL021W	1.957	URA3; enzyme involved in biosynthesis of pyrimidines
RDN25-1	1.887	25S rRNA

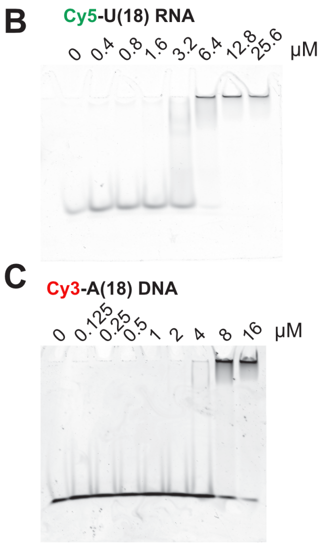

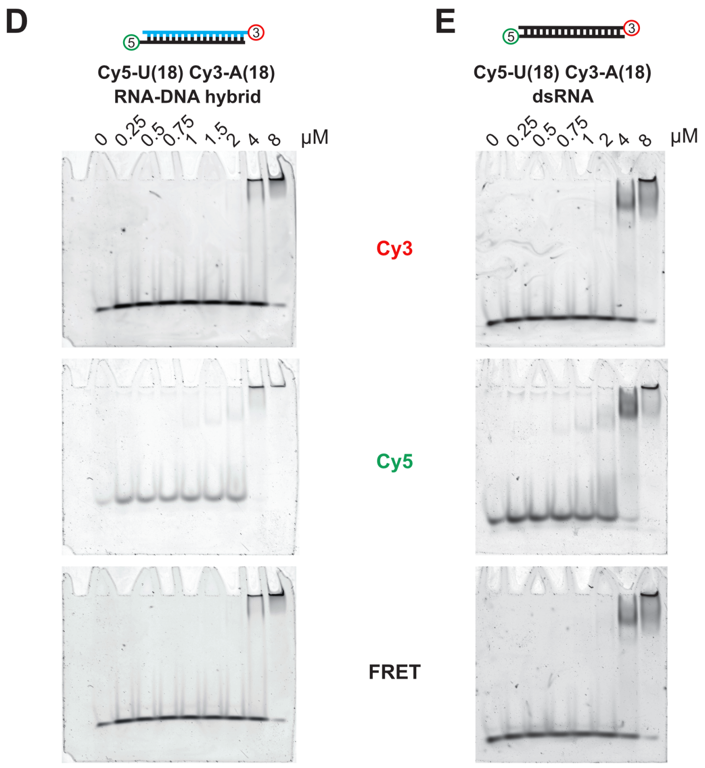

Figure S1: Unspecific RNA binding by Loc1p. (A) Microarray analysis of RNAs immunoprecipitated with TAP-tagged Loc1p revealed binding to rRNAs of the large ribosomal subunit. This table includes the ten most abundant features (including ORF and ncRNAs) that were detected in at least two out of three immunoprecipitations. Microarray data is available for download via the PUMA database (http://puma.princeton.edu), experiment set No. 7342. **(B-E)** Loc1p binds unspecifically to single-stranded and double-stranded nucleic acids. EMSAs with fluorescence-labeled nucleic acids show that Loc1p binds to single-stranded RNA (B) with a similar affinity as it binds to single-stranded DNA (C). In-gel FRET experiments (D,E) show that Loc1p binds to double-stranded RNA as well as to an RNA-DNA hybrid. For fluorescence EMSAs 100 nM of labeled RNA or DNA and the indicated amount of MBP-Loc1p were incubated at RT for 25 min before complexes were resolved on a 6 % TBE-PAGE. Fluorescence signals were detected using a typhoon scanner.

No	Sequence	Binding	(R,K) / (R,K,D,E)	Motif
A1	MAPKKPSKRQNLRREVAPEV	++	75%	+
A2	KKPSKRQNLRREVAPEVFQD	++	67%	+
A3	SKRQNLRREVAPEVFQDSQA	++	57%	
A4	QNLRREVAPEVFQDSQARNQ		50%	
A5	RREVAPEVFQDSQARNQLAN		50%	
A6	VAPEVFQDSQARNQLANVPH		33%	
A7	EVFQDSQARNQLANVPHLTE		25%	
A8	QDSQARNQLANVPHLTEKSA		50%	
A9	QARNQLANVPHLTEKSAQRK	+++	80%	
A10	NQLANVPHLTEKSAQRKPSK	+++	80%	+
A11	ANVPHLTEKSAQRKPSKTKV	+++	83%	+
A12	PHLTEKSAQRKPSKTKVKKE	+++	78%	+
A13	TEKSAQRKPSKTKVKKEQSL	+++	78%	+
A14	SAQRKPSKTKVKKEQSLARL	+++	86%	
A15	RKPSKTKVKKEQSLARLYGA	+++	88%	
A16	SKTKVKKEQSLARLYGAKKD	+++	78%	
A17	KVKKEQSLARLYGAKKDKKG	+++	78%	
A18	KEQSLARLYGAKKDKKGKYS	+++	78%	+
A19	SLARLYGAKKDKKGKYSEKD	+++	70%	+
A20	RLYGAKKDKKGKYSEKDLNI	++	70%	+
B1	GAKKDKKGKYSEKDLNIPTL	++	67%	+
B2	KDKKGKYSEKDLNIPTLNRA	+++	67%	+
B3	KGKYSEKDLNIPTLNRAIVP	++	67%	
B4	YSEKDLNIPTLNRAIVPGVK	+	75%	
B5	KDLNIPTLNRAIVPGVKIRR	+++	83%	
B6	NIPTLNRAIVPGVKIRRGKK	+++	100%	+
B7	TLNRAIVPGVKIRRGKKGKK	+++	100%	+
B8	RAIVPGVKIRRGKKGKKFIA	+++	100%	+
B9	VPGVKIRRGKKGKKFIADND	+++	78%	+
B10	VKIRRGKKGKKFIADNDTLT	+++	78%	+
B11	RRGKKGKKFIADNDTLTLNR	+++	78%	+
B12	KKGKKFIADNDTLTLNRLIT	+++	71%	
B13	KKFIADNDTLTLNRLITTIG		75%	
B14	IADNDTLTLNRLITTIGDKY		40%	
B15	NDTLTLNRLITTIGDKYDDI		33%	
B16	LTLNRLITTIGDKYDDIAES		33%	
B17	NRLITTIGDKYDDIAESKLE		38%	
B18	ITTIGDKYDDIAESKLEKAR		44%	
B19	IGDKYDDIAESKLEKARRLE		45%	
B20	KYDDIAESKLEKARRLEEIR	+	50%	
C1	DIAESKLEKARRLEEIRELK		50%	
C2	ESKLEKARRLEEIRELKRKE	+	57%	
C3	LEKARRLEEIRELKRKEIER	+	57%	+
C4	ARRLEEIRELKRKEIERKEA	+	57%	+
C5	LEEIRELKRKEIERKEALKQ	+	54%	+
C6	IRELKRKEIERKEALKQDKL	++	62%	+
C7	LKRKEIERKEALKQDKLEEK	++	57%	+
C8	KEIERKEALKQDKLEEKKDE		47%	+
C9	ERKEALKQDKLEEKKDEIKK		53%	+
C10	EALKQDKLEEKKDEIKKKSS	+	57%	+
C11	KQDKLEEKKDEIKKKSSVAR	++	62%	+
C12	KLEEKKDEIKKKSSVARTIR	++	67%	+
C13	EKKDEIKKKSSVARTIRRKN	+++	75%	
C14	DEIKKKSSVARTIRRKNKRD	++	75%	+
C15	KKKSSVARTIRRKNKRDMLK	+++	91%	+
C16	SSVARTIRRKNKRDMLKSEA	+++	78%	+
C17	ARTIRRKNKRDMLKSEAKAS	++	80%	+
C18	IRRKNKRDMLKSEAKASESK	+++	73%	+
C19	KNKRDMLKSEAKASESKTEG	+	60%	+
C20	RDMLKSEAKASESKTEGRKV	+	60	
D1	LKSEAKASESKTEGRKVKKV	+++	70%	+
D2	EAKASESKTEGRKVKKVSFA	+++	67%	+
D3	AKASESKTEGRKVKKVSFAQ	++	75%	+

normalized intensity	Binding
10 - 39 %	+
40 - 69 %	++
70 - 100 %	+++

Figure S2: **Peptides used in the peptide tiling array and quantified binding intensities.** Analysis of *ASH1* E3 binding to the Loc1p peptide tiling array. Shown are the position in the array, the peptide sequence, normalized binding intensity for each position, ratio of positively charged residues versus charged residues, and whether the respective peptide contains a repetitive, positively charged motif. The peptide with the highest intensity was set to 100 % and other peptides were normalized accordingly. Peptides with a higher portion of negatively charged residues show lower intensities and thus binding.

A

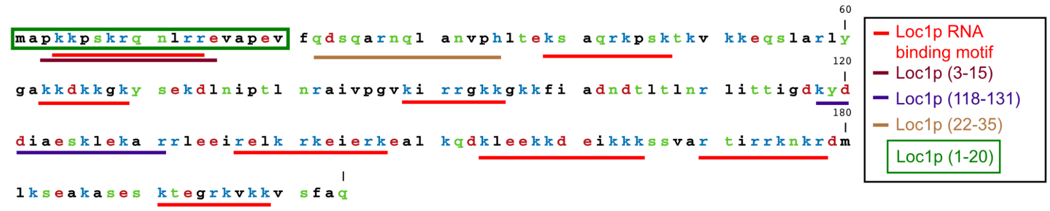

B

Loc1p: biochemical data	
Amino acids	204
Molecular weight	23.6 kDa
Isoelectric point	10.3
No of + charged residues	61
No of - charged residues	30

C

Name	Sequence	motif	Assay
Loc1p (3-15)	pkkpskrqnlrre	1	NMR
Loc1p (118-131)	kyddiaesklekar	0	NMR
Loc1p (22-35)	qdsqarnqlanvph	0	NMR
Loc1p (1-20)	mapkkpskrqnlrrevapev	1	Chaperone assay
Loc1p $(1\text{-}20)_2$	mapkkpskrqnlrrevapevmapkkpskrqnlrrevapev	2	Chaperone assay

D

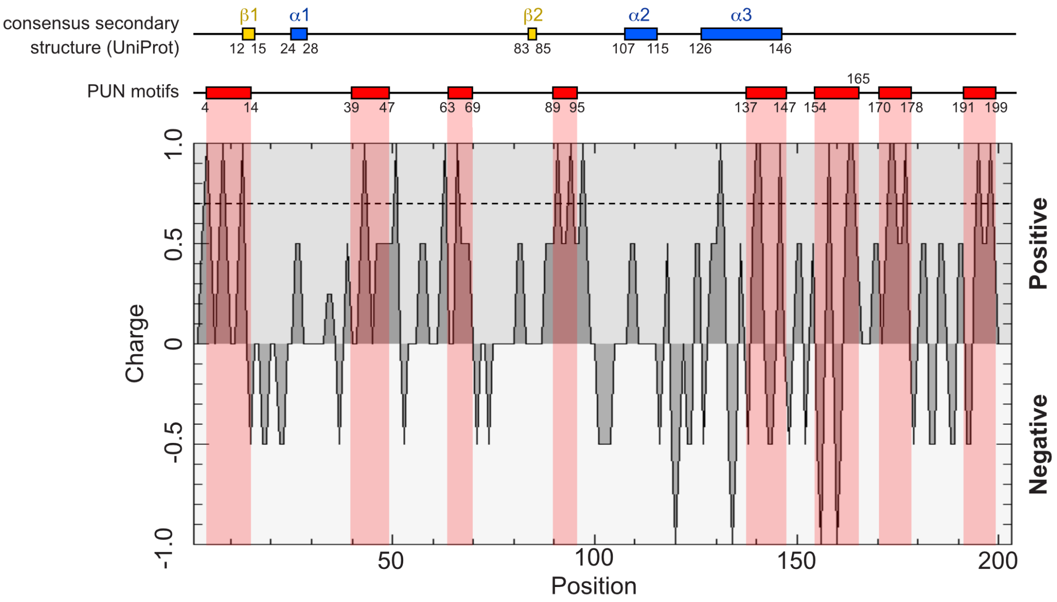

Figure S3: Protein regions of Loc1p interacting with RNA and PUN motifs. **(A)** Amino acid sequence of Loc1p colored according to their electrochemical properties (charges: negative in red, positive in blue, polar neutral in green, polar hydrophobic in black). RNA binding motifs within the Loc1p sequence are underlined in red. **(B)** Loc1p protein parameters derived from www.expasy.org/proteomics. **(C)** List Loc1p-derived peptides used in this study. The RNA binding motif is depicted in red and the experiment is indicated. The sequences of the used peptides are also indicated in (B). **(D)** Charge plot of the Loc1p sequence. The charge is plotted against the amino acid sequence of Loc1p (window size: 2 amino acids). Above the charge plot, schematic representations of the Loc1p sequence illustrate the location of secondary structure elements (as described in UniProt) and PUN repeats that match the consensus sequence. Note that each PUN repeat is characterized by positively charged amino acids while the spacers typically lack positive charges and instead bear one or more negatively charged residues.

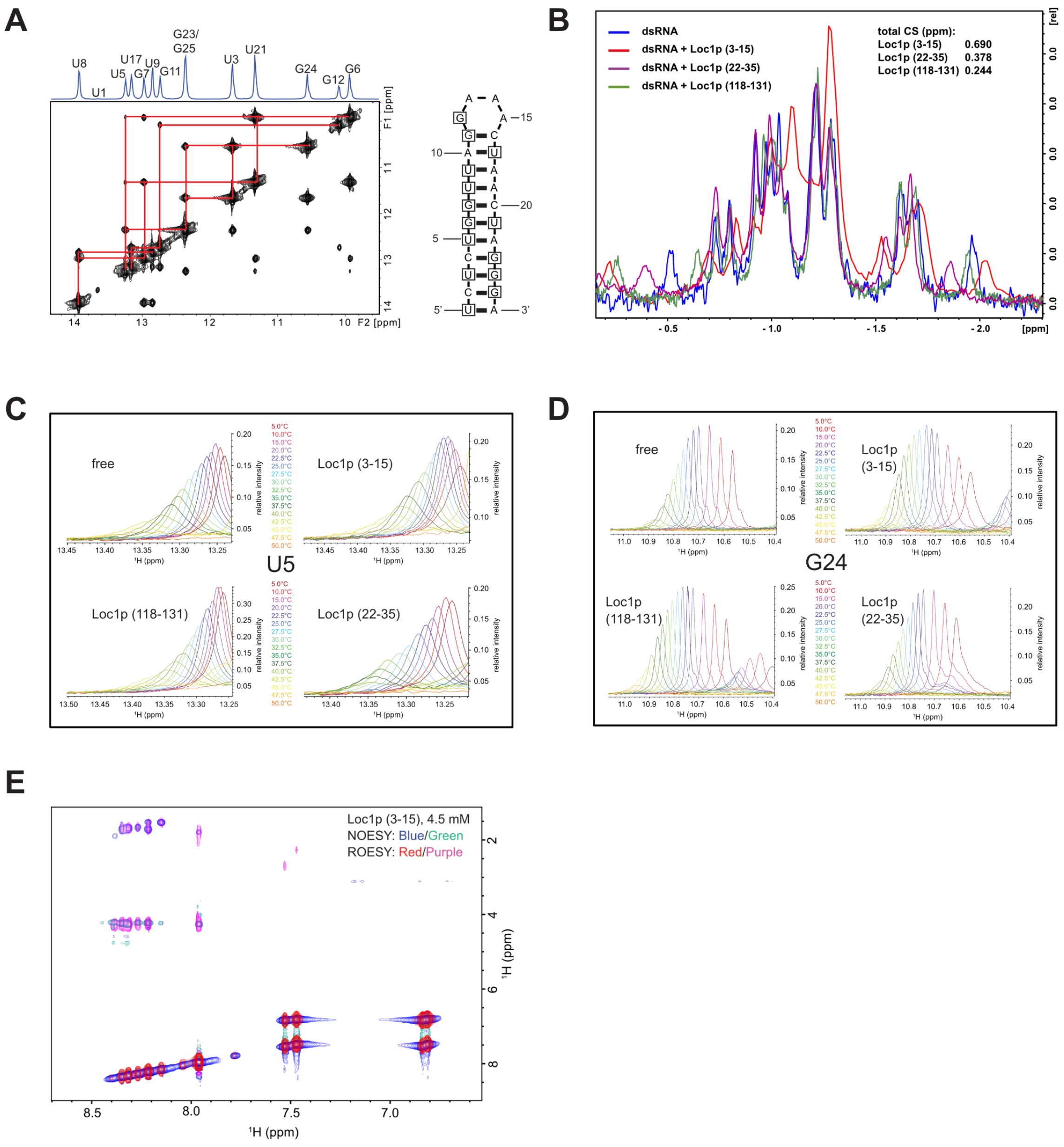

Figure S4: NMR studies on the interaction between Loc1p and RNA. (A) Complete imino signal assignment of the dsRNA. To the right the stem-loop is shown with assigned bases marked with a square. **(B)** Titration of all three peptides in 1D-^{31}P-NMR experiments with stem loop RNA. Although each peptide resulted in resonance shifts at high concentrations, they showed different effects. Resonances of phosphodiester bonds located in an A-type or B-type nucleic acid helix usually cluster around chemical shifts of -1 ppm, which can also be observed here (Gorenstein, 1984; Wüthrich, 1986). Terminal phoshomonoesters are shifted downfield and phosphodiesters of bases located at a terminal stem close to a loop are shifted upfield. The resonances (at -0.5 and -2 ppm) corresponding to terminally located phosphorus atoms exhibit the strongest shifts upon addition of peptides (5:1 ratio, peptide:RNA). Interestingly, Loc1p (22-35) and Loc1p (118-131) do not induce shifts on resonances clustered around -1 ppm, but Loc1p (3-15) does, indicating that Loc1p (3-15), the only one with chaperone activity and showing to stabilize the RNA strongest also binds to the backbone of nucleotides located at the core of the helix. **(C, D)** Selected, overlayed 1D imino NMR experiments of the RNA tetraloop free and bound to different peptides during temperature scans. (C) Zooms into the spectral

region of the U5 imino peak of spectra of free RNA (upper left), bound to Loc1p(3-15) (upper right), Loc1p(118-131) (lower left), and Loc1p(22-35) (lower right). The peak positions have been altered manually to allow for a better comparison between signal intensities. The peak intensities for each temperature have been used to generate the melting curves and to estimate the melting temperature for U5 base pairing. Loc1p(3-15) stabilizes this base pair compared to free RNA, as the peak is still at its maxima at 25°C compared to 15°C in absence of peptide. The other two peptides on the contrary destabilize this base pair and signal intensity already decreases after raising the temperature above 10°C. (D) shows the same as (C) but for the imino peak of G24. This peak was more isolated and it was not necessary to adjust the peak position manually. Thus, their chemical shifts are the experimental ones. The color and corresponding temperatures are shown in between each spectrum. **(E)** Comparison of 2D ROESY and NOESY NMR spectra of Loc1p(3-15). In order to assess whether the lack of NOEs for Loc1p(3-15) in absence of RNA is due to lack of structure or due to its size and thus being at the zero-crossing for NOESY-based experiments, we compared a ROESY (no zero-crossing) and NOESY spectra. Even at 4.5 mM concentration, also the ROESY experiment shows only a few intra-residual NOE cross peaks, clearly demonstrating that Loc1p(3-15) is unstructured.

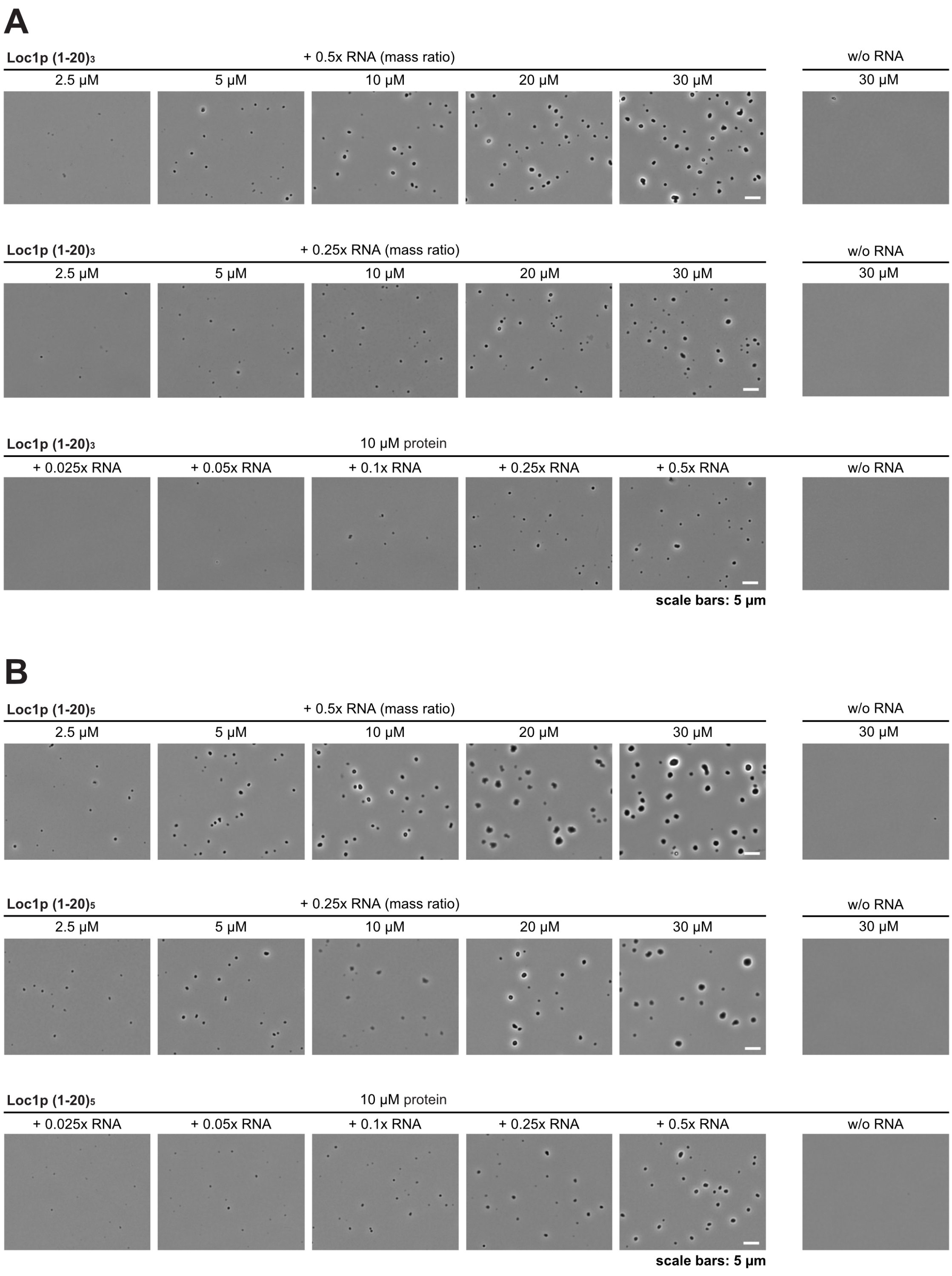

Figure S5: Assessment of condensate formation by different PUN-motif containing proteins. (A-B) RNA-dependent condensate formation by protein fragments containing three (A) or five (B) copies of the most N-terminal PUN motif of Loc1p. Condensate formation occurred already at lower concentrations when Loc1p $(1\text{-}20)_5$ was used instead of Loc1p $(1\text{-}20)_3$. No phase separation was observed in absence of RNA. The formation of condensates was assessed by phase-contrast microscopy.

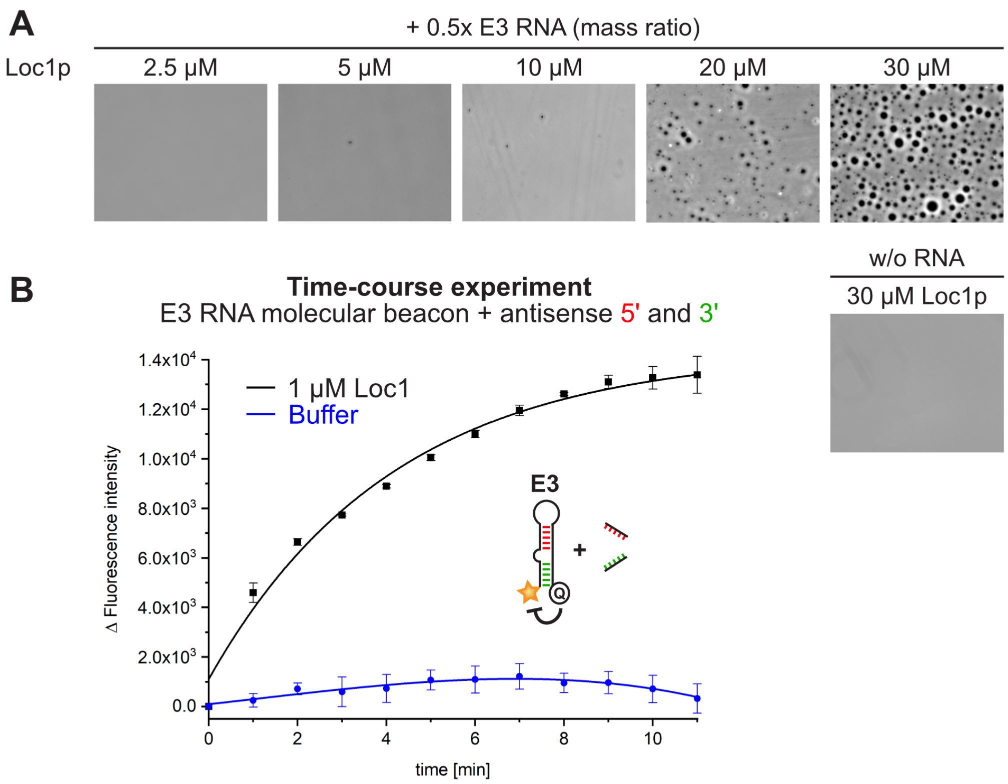

Figure S6: Loc1p forms condensates with a 28nt E3 localization element (LE) of *ASH1* mRNA and promotes conformational changes in its stem-loop. (A) Phase separation of full-length Loc1p is observed at concentrations of 5 µM or higher when mixed with the 28 nt E3 LE stem-loop, while no phase separation is observed without RNA. **(B)** Loc1p promotes conformational changes in the stem-loop of a 28nt E3 LE molecular beacon (5'-FAM and 3'-BMNQ-535). In the presence of two 5nt antisense RNAs (5'anti-E3 and 3'anti-E3), titration of Loc1p changes the fluorescence intensity of an E3 LE molecular beacon in time-course experiments, suggesting that Loc1p catalyzes the formation of the energetically most favored state.

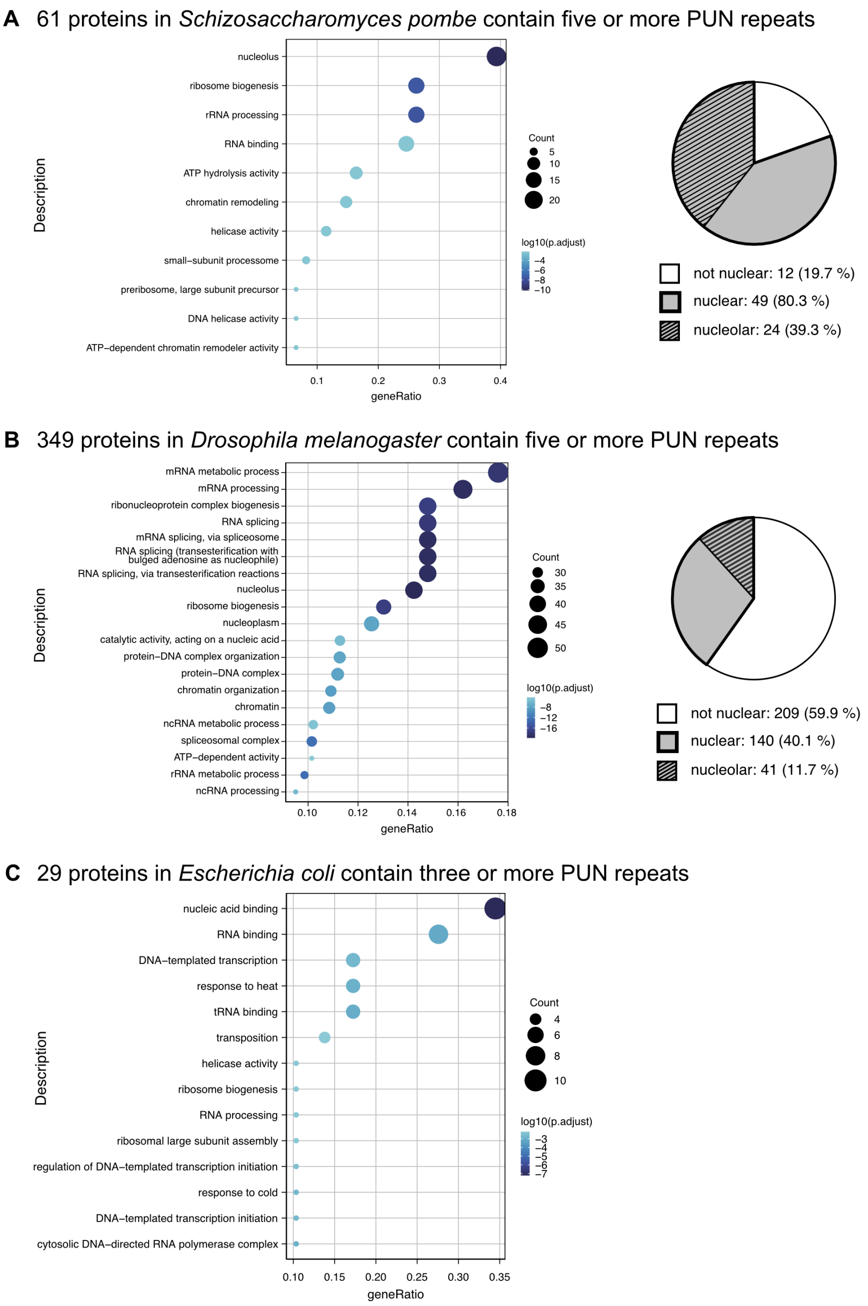

Figure S7: Enrichment of PUN motif-containing proteins in distant eukaryotic and procaryotic species. (A) GO-term analysis of 61 *Schizosaccharomyces pombe* proteins containing at least five PUN motifs (three of them in 150 aa) shows that pathways of ribosome biogenesis, RNA processing, and RNA binding are enriched. Analysis of the cellular localization of the 61 hits shows a clear preference for the nuclear and nucleolar compartments of the cell. **(B)** 349 *Drosophila melanogaster* proteins containing five or more PUN motifs with three motifs in at least 150 aa are also enriched in the nucleus. GO-term analysis of the cellular localization of the 349 hits shows a preference for the nuclear and nucleolar compartments of the cell. **(C)** We identified 29 *Escherichia coli* proteins containing at least 3 PUN motifs, which are involved in RNA metabolic processes. GO-term analysis of annotated processes shows that pathways of nucleic acid binding and transcription are enriched.

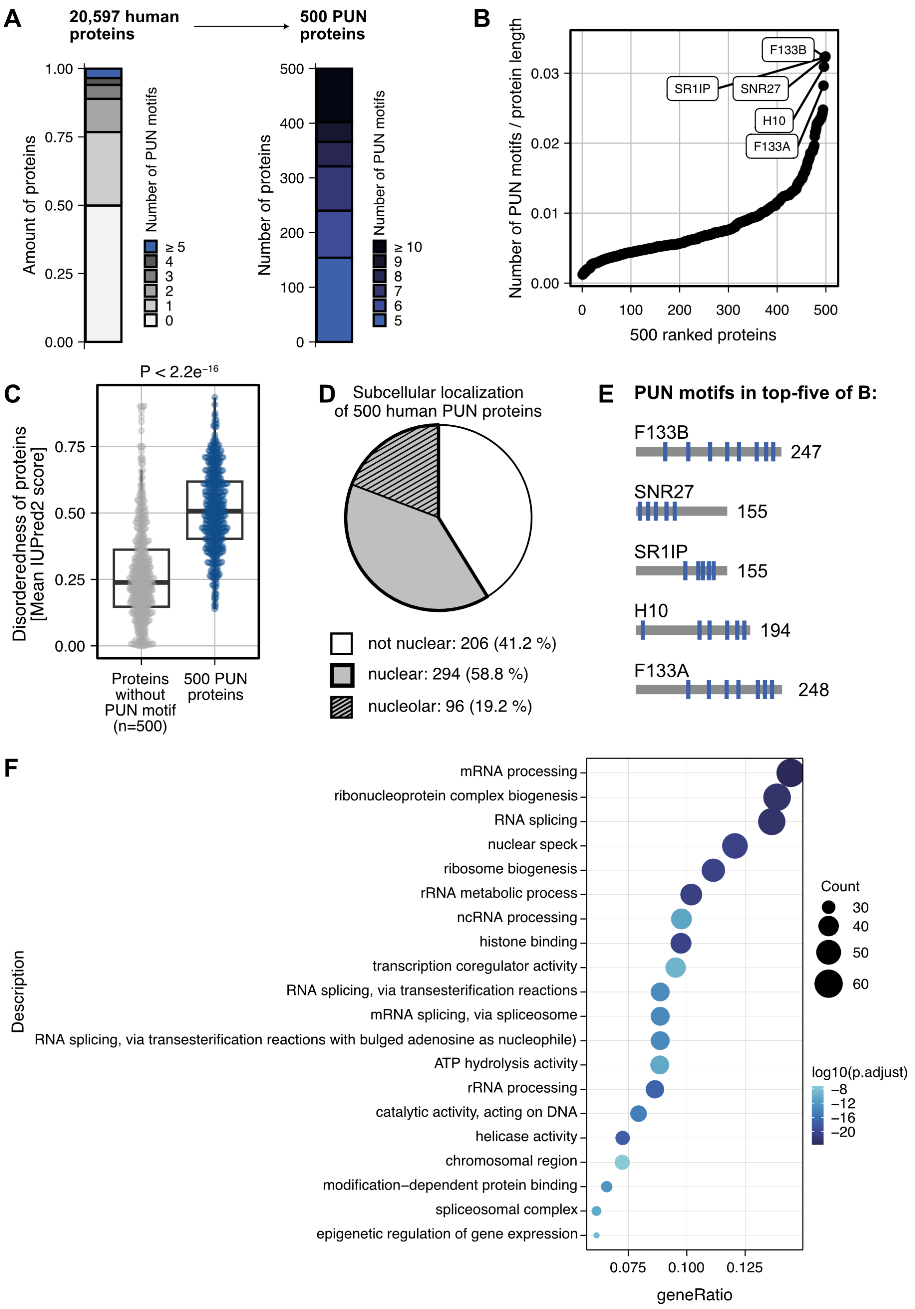

Figure S8: Human PUN motif-containing proteins are enriched in the nucleus and involved in RNA-related processes. (A) Identification of 500 human proteins that contain at least five PUN motifs in total and three in 150 amino acids. **(B)** PUN motif density in the 500 human PUN proteins. Proteins with the highest PUN motif density are involved in RNA-binding, DNA condensation, or splicing. The sperm head cytoskeletal calyx protein CYLC2 was removed because of its involvement in an RNA-unrelated process. **(C)** Human PUN proteins are characterized by a higher degree of disorderedness compared to proteins that do not match the criteria, reflecting the preference of PUN motifs for disordered regions. **(D)** Analysis of the intracellular distribution of the 500 hits shows a clear preference for the nuclear compartment of the cell (59 %). **(E)** Distribution of PUN motifs in top-hits from (B). **(F)** GO-term analysis of annotated processes ordered by P-values. The RNA processing pathways are enriched in human proteins bearing PUN motifs.

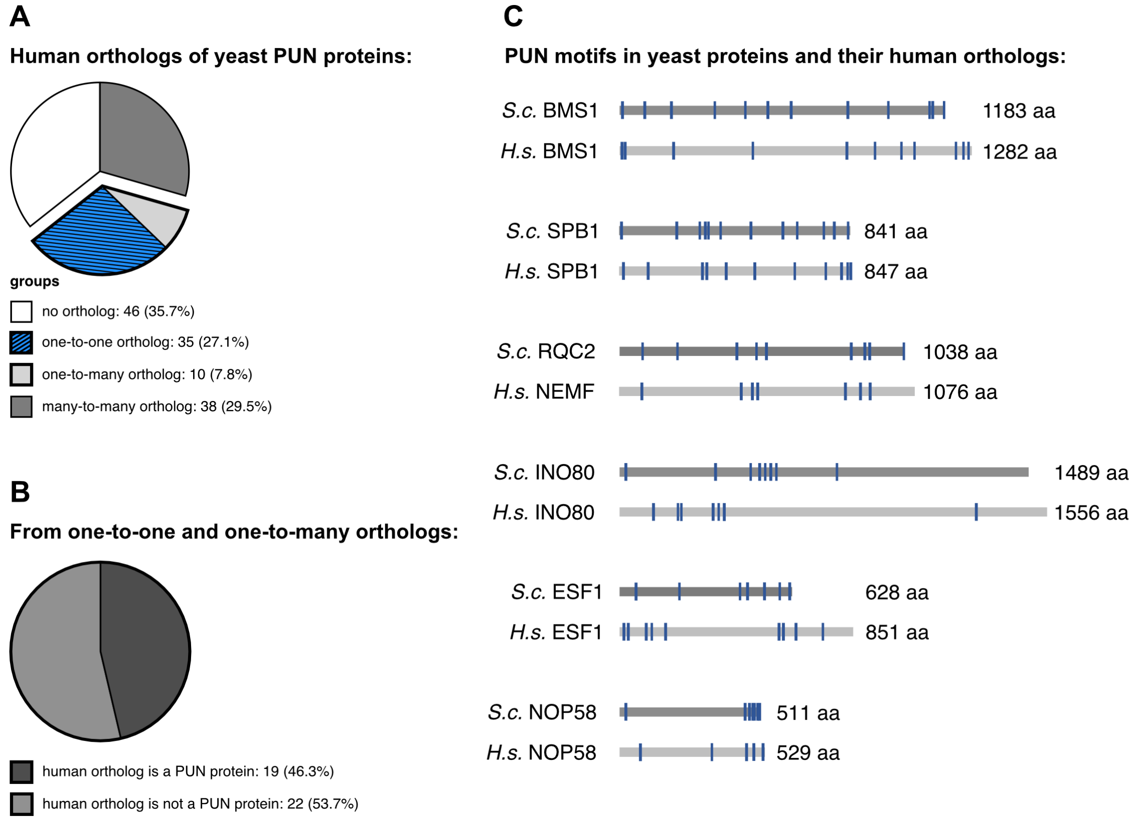

Figure S9: Characterization of human orthologs of the 95 *S. cerevisiae* PUN proteins. (A) A database search for human orthologs using the 95 yeast PUN proteins identified 35 one-to-one and 10 one-to-many orthologs, while 46 proteins have no human counterpart. **(B)** 46% of the one-to-one and one-to-many human orthologs are also PUN proteins in human, while 53% do not fulfill both criteria (5 or more PUN motifs and at least 3 PUN motifs within 150 amino acids). **(C)** Examples from the top-20 of yeast PUN proteins with their human orthologs. Protein length (in amino acids) is shown to scale in bar representation and the relative locations of PUN motifs are indicated by blue rectangles. Note that orthologous proteins are similar in length and clustering of the PUN motifs.

Table S1: **Isoelectric points of ribosomal proteins**, Theoretical isoelectric point calculated with ProtParam (www.expasy.org). Proteins with pI ≥ 9.0 are shown in green, between 8.9 and 7.0 in yellow (none) and with pI ≤ 6.9 in red.

Large subunit	Size (aa)	pI
RPL1A/B	217	10.51
RPL2A/B	254	11.74
RPL3	387	11.1
RPL4A/B	362	11.4
RPL5	297	6.82
RPL6A	176	10.91
RPL6B	176	10.89
RPL7A/B	244	10.97
RPL8A	256	10.85
RPL8B	256	10.83
RPL9A	191	10.54
RPL9B	191	10.47
RPL10	221	10.84
RPL11A/B	174	10.73
RPL12A/B	165	10.2
RPL13A	199	11.79
RPL13B	199	11.71
RPL14A/B	138	11.63
RPL15	204	11.96
RPL16A	199	11.28
RPL16B	198	11.34
RPL17A/B	184	11.62
RPL18A/B	186	12.24
RPL19A/B	189	11.93
RPL20A/B	172	11.12
RPL21A/B	160	11.2
RPL22A	121	6.01
RPL22B	122	5.98
RPL23A/B	137	11.11
RPL24A	155	11.9
RPL24B	155	11.97
RPL25	142	10.94
RPL26A	127	11.35
RPL26B	127	11.25
RPL27A/B	136	11.16
RPL28	149	11.4
RPL29	59	11.99
RPL30	105	10.64
RPL31A/B	113	10.8
RPL32	130	11.83
RPL33A/B	107	11.76
RPL34A/B	121	11.56
RPL35A/B	120	11.36
RPL36A/B	100	12.15
RPL37A	88	12.19
RPL37B	88	12.32
RPL38	78	11.65
RPL39	51	12.55
RPL40A/B	128	10.63
RPL41A/B	25	12.96
RPL42A/B	106	11.38
RPL43A/B	92	11.21

Small subunit	Size (aa)	pI
RPS0A	252	4.47
RPS0B	252	4.51
RPS1A	255	10.79
RPS1B	255	10.81
RPS2	254	11.21
RPS3	240	10.22
RPS4A/B	261	10.9
RPS5	225	9.11
RPS6A/B	236	11.22
RPS7A	190	10.64
RPS7B	190	10.73
RPS8A/B	200	11.42
RPS9A	197	10.8
RPS9B	195	10.9
RPS10A	105	9.63
RPS10B	105	9.92
RPS11A/B	156	11.55
RPS12	143	4.5
RPS13	151	11.24
RPS14A	137	11.43
RPS14B	138	11.28
RPS15	142	11.45
RPS16A/B	143	11.08
RPS17A/B	136	11.28
RPS18A/B	146	11.06
RPS19A/B	144	10.45
RPS20	121	10.33
RPS21A/B	87	5.9
RPS22A/B	130	10.76
RPS23A/B	145	11.47
RPS24A/B	135	11.29
RPS25A/B	108	11.13
RPS26A	119	11.49
RPS26B	119	10.6
RPS27A/B	82	9.51
RPS28A/B	67	11.42
RPS29A	56	11.06
RPS29B	56	10.8
RPS30A/B	63	12.24
RPS31	152	10.67

Stalk	Size (AA)	pI
RPP0	312	4.56
RPP1A	106	3.61
RPP1B	106	3.68
RPP2A	106	3.77
RPP2B	110	3.89
RKM2	479	5.1

Table S2: **Microarray data showing Loc1p-bound RNAs.** List of most enriched RNAs when comparing mean of three Loc1p experiments with corresponding control experiments. YORF descriptions starting with "SPL2" refer to probes against intronic regions of a given gene (named thereafter), descriptions starting with "I" refer to probes against intergenic regions. The full set of dataset is available for download via the PUMA database (http://puma.princeton.edu; experiment #7342).

YORF	Description	Loc1p #1	Loc1p #2	Loc1p #3	Mean Loc1p	Rank	Mean wt
SPL2YML085C	TUB1; alpha-tubulin	3,88			3,880	1,000	0,745
RDN58-1	5.8S rRNA	5,41	2,13	3,87	3,803	0,999	0,380
IYLR161W	encoded near the ribosomal DNA region	5,57	1,64	3,91	3,707	0,999	3,233
RDN5-1	5S rRNA	5,39	1,21	3,5	3,367	0,999	2,565
IYLR159W	encoded near the ribosomal DNA region	5,01	0,96	3,57	3,180	0,999	3,575
SPL2YML025C	YML6; mitochondrial ribosomal protein	2,92			2,920	0,999	0,525
SPL2YLR406C	RPL31B; large ribosomal subunit protein	2,66			2,660	0,999	0,545
SPL2YJL225C	uncharacterized ORF	2,65			2,650	0,998	0,630
YNL204C	protein of unknown function			2,5	2,500	0,998	0,110
SPL2YIL052C	RPL34B; large ribosomal subunit protein	3,64		0,75	2,195	0,998	0,678
YDR403W	Sporulation-specific enzyme		1,18	3,03	2,105	0,998	0,028
YKL084W	HOT13; mitochondrial protein	1,75	0,94	3,55	2,080	0,998	-0,300
YHL041W	dubious ORF	1,59	2,19	2,39	2,057	0,998	-0,158
YGL183C	MND1; required for recombination		2,01		2,010	0,997	0,633
YOR343C	dubious ORF			2,01	2,010	0,997	0,060
YLR119W	SRN2; ESCRT-I component	0,93	1,92	3,17	2,007	0,997	-0,435
SPL2YIL004C	BET1; v-SNARE	3,08		0,93	2,005	0,997	0,530
YJL137C	GLG2; Glycogenin glucosyltransferase		1,6	2,36	1,980	0,997	-0,015
SPL2YMR225C	MRPL44; mitochondrial ribosomal protein	2,98		0,98	1,980	0,997	0,403
YCR018C	SRD1; processing of pre-rRNA	2,93	1,62	1,38	1,977	0,997	-0,060
YEL021W	URA3; OMP decarboxylase	2,1	1,39	2,38	1,957	0,997	0,208
YPR096C	protein of unknown function		0,55	3,34	1,945	0,996	0,070
YJL103C	protein of unknown function		1,89		1,890	0,996	0,293
RDN25-1	25S rRNA	2,62	1,4	1,64	1,887	0,996	-1,288
IYLRCDELTA8	Ty1 long terminal repeat	3,31	-0,38	2,71	1,880	0,996	3,253
YOR391C	HSP33; chaperone		1,18	2,56	1,870	0,996	0,088
SPL2YLR078C	BOS1; v-SNARE	1,54	1,05	3,02	1,870	0,996	0,565
YJL135W	dubious ORF		1,78	1,89	1,835	0,995	0,070
YGR217W	CCH1; calcium channel	2,9	0,81	1,79	1,833	0,995	-0,240
YJL199C	dubious ORF	1,64	1,91	1,91	1,820	0,995	0,000

Table S5: **Data collection and refinement statistics for the RNA hairpin tetraloop crystal structure**. Values in parentheses are for the highest-resolution shell.

**Data collection**	
Beamline	SLS PXIII X06DA
Wavelength (Å)	1.0000
Space group	$P2_12_12_1$
Cell dimensions a, b, c (Å)	24.27, 52.43, 107.18
Resolution (Å)	50 – 2.0 (2.05 – 2.00)
R_{merge} (%)	9.2 (56.4)
I / σI	13.1 (3.49)
CC (1/2)	0.987 (0.681)
Completeness (%)	97.0 (96.0)
Redundancy	5.9 (5.7)
**Refinement**	
Resolution (Å)	2.0
No. reflections	9,048
R_{work} / R_{free} (%)	17.5 / 24.1
No. atoms	
RNA	1,108
Ba	10
Water	97
B-factor overall (Å^2)	31.5
R.m.s. deviations	
Bond lengths (Å)	0.013
Bond angles (°)	2.28
PDB ID	6YMC

External Tables for download:

Table S3: Microarray data showing all Loc1p-bound RNAs.

Table S4: List of nucleic acids used in this study.

Table S6: PUN motif proteins in *S. cerevisiae*.

Table S7: PUN motif proteins in *S. pombe*.

Table S8: PUN motif proteins in *D. melanogaster*.

Table S9: PUN motif proteins in *E. coli*.

Table S10: PUN motif proteins in *H. sapiens*.

Table S11: *S. cerevisiae* PUN motif proteins with their *H. sapiens* orthologs.

Movie S1: Time recording of Loc1p/RNA LLPS fusion.

Movie S2: Time recording of photobleaching and fluorescence recovery in a Loc1p/RNA LLPS FRAP experiment.
